# HTLV-3 and HTLV-4 antisense proteins activate JunB-, c-Jun- and JunD-dependent transcription

**DOI:** 10.1186/1742-4690-8-S1-A141

**Published:** 2011-06-06

**Authors:** Émilie Larocque, Guy Lemay, Jean-Michel Mesnard, William M Switzer, Benoit Barbeau

**Affiliations:** 1 Départements des sciences biologiques, Université du Québec à Montréal, Montréal, Canada, H2X 3Y5; 2 Départements de microbiologie et immunologie, Université de Montréal, Montréal, Canada, H3T 1J4; 3 Centre des Études des Agents Pathogènes et Biotechnologies pour la Santé (CPBS), Université Montpellier 1, CNRS/UM1/UM2 UMR 5236, Montpellier, France; 4 Division of HIV/AIDS Prevention, National Center for HIV, AIDS, Viral Hepatitis, STD, and TB prevention, Centers for Disease Control and Prevention, Atlanta, GA, 30333, USA

## Background

The antisense transcript-encoded HBZ protein acts upon viral and cellular gene expression through its modulation of the activation of transcription factors such as Jun family members. A possible link between HBZ and ATL development has also been highlighted. HTLV-3 and HTLV-4 are two newly discovered human retroviruses closely related to HTLV-1; no known diseases have yet been associated to these viruses. Based on previous in silico analyses, our aim was to test for the presence of antisense transcripts in these new viruses and to functionally assess these viral proteins.

## Results

By RT-PCR analyses, HTLV-3 and HTLV-4 were shown to produce spliced antisense transcripts termed APH (Antisense Protein of HTLV)-3 and APH-4. Confocal microscopy analyses of cells expressing Myc- or GFP-tagged APH-3 and APH-4 showed distinct localization but partial colocalization with HBZ to the nucleus and the nucleoli. Using LTR-luciferase constructs, we demonstrated that APH-3 and APH-4 inhibited Tax1 and Tax3 transactivation of respective LTRs. In transfection experiments with a collagenase promoter-driven luciferase reporter construct, we showed that APH-3 and APH-4 modulated JunB- and c-Jun-dependent transcription differently from HBZ. Deletion mutants indicated that this upregulation of the transactivation potential of Jun factors was mediated through the atypical bZIP domain of APH-3 and APH-4.

## Conclusion

We show that HTLV-3 and HTLV-4 express new viral proteins, APH-3 and APH-4, which modulate Jun-dependent transcription differently from HBZ. These data underscore the importance of our study on APH-3 and APH-4 to help in better understanding the role of HBZ in the development of ATL.

